# BIPAD: A web server for modeling bipartite sequence elements

**DOI:** 10.1186/1471-2105-7-76

**Published:** 2006-02-17

**Authors:** Chengpeng Bi, Peter K Rogan

**Affiliations:** 1 Laboratory of Human Molecular Genetics, Children's Mercy Hospital & Clinics, 2401 Gillham Road, Kansas City, MO 64108, USA; 2 School of Computer Science and Engineering, University of Missouri-Kansas City, 5115 Oak St., MO 64110, USA

## Abstract

**Background:**

Many dimeric protein complexes bind cooperatively to families of bipartite nucleic acid sequence elements, which consist of pairs of conserved half-site sequences separated by intervening distances that vary among individual sites.

**Results:**

We introduce the Bipad Server [1], a web interface to predict sequence elements embedded within unaligned sequences. Either a bipartite model, consisting of a pair of one-block position weight matrices (PWM's) with a gap distribution, or a single PWM matrix for contiguous single block motifs may be produced. The Bipad program performs multiple local alignment by entropy minimization and cyclic refinement using a stochastic greedy search strategy. The best models are refined by maximizing incremental information contents among a set of potential models with varying half site and gap lengths.

**Conclusion:**

The web service generates information positional weight matrices, identifies binding site motifs, graphically represents the set of discovered elements as a sequence logo, and depicts the gap distribution as a histogram. Server performance was evaluated by generating a collection of bipartite models for distinct DNA binding proteins.

## Background

Dimeric transcription factors often bind to bipartite genomic sequence elements (TFBS) in promoters, which are composed of two adjacent degenerate motifs with four possible orientations, separated by a flexible nucleotide spacer of unspecified sequence [2,3]. For example, nuclear receptor transcription factors, which form homo- or heterodimeric complexes, can potentiate transcription of downstream target genes by binding of degenerate bipartite sites that display partial internal sequence symmetry [4]. Characterization of these motifs, locating these sites, orientations and estimation of their binding affinities is crucial to understanding transcriptional responses to developmental and environmental cues.

Bipartite sequence patterns can be discovered by *de novo *methods that enumerate, such as spaced dyad [5] and structured motif [6,7] algorithms, and with position weight matrices (PWM), such as those used by BioProspector [8] and Bipad [2,9]. Given a set of unaligned DNA sequences sharing a common bipartite or single-block pattern, the Bipad algorithm finds such patterns that maximizes total information content [10], which is computed from the information contents for left- and right motifs, and a gap penalty based on the surprisal function. The site information contents are related to their binding strengths [10,11], which can then be verified in the laboratory [12]. Bipad simultaneously searches each of the four possible types of orientations (see below) for a given bipartite pattern. The single-block motif can be treated as a bipartite pattern with zero length nucleotide gap between half-sites. Bipad outputs two PWM matrices for half-site models and associated gap distribution for a bipartite pattern search or one PWM matrix for one-block motif. Using a stochastic greedy search strategy driven by a set of randomly seeds (bipartite coordinates), Bipad performs multiple local alignment and cyclic refinement of the search operation for a specified number of cycles. Additional cycles lead the search toward the preferred solution and reduce the likelihood of producing inferior alignments that may arise during a single cycle.

Bipad performed equivalently or better in both *de novo *single-block and bipartite motif discovery, particularly for sites with conserved binding sequences that are present on both strands [2]. We benchmarked Bipad against other popular *de novo *local alignment software, including GLAM, Gibbs Sampler, and CONSENSUS, using experimentally-verified *E. coli *CRP binding sites. Bipad exhibited better sensitivity and specificity than Gibbs and CONSENSUS and results equivalent to those obtained with GLAM [2]. For bipartite motif discovery, we compared Bipad with BioProspector [8]. Bipad is uniquely designed to recognize binding sites on either strand (in all four potential orientations), increasing its sensitivity for detection of reverse direct and inverted half sites [2]. Unlike BioProspector, Bipad assumes a uniform underlying genomic composition. The objective function is thus reduced to one that minimizes Shannon entropy, which simplifies the mathematical model and accelerates numerical computation. The convergence in each cycle is therefore very rapid; this property supports implementation of Bipad as a distributed computational process that will be especially useful in aligning large datasets. Indeed, Bipad generalizes the bipartite motif discovery problem to allow any range of gap lengths to be specified and permits the two half motifs to be either homogeneous (perfect or imperfect repetitive half sites) or heterogeneous (i.e. different patterns or motif widths of two half sites).

However, multiple local alignment algorithms such as Bipad are susceptible to producing sub-optimal alignments that result from detection of local minima of the objective function, rather than producing a global optimal alignment. Bipad avoids local minima by running a specified number of cycles, with each cycle initiating with different sets of binding site coordinates. This stochastic cycling strategy has proven to be efficient, but it does not ensure a global optimum [2].

The Bipad web server performs sequence pattern discovery of functionally-related DNA sequences, typically containing binding sites recognized by a cognate protein(s), embedded within a heterogeneous sequence background. The public web interface, which is written in Perl, executes the Bipad program. The Web program displays the bipartite (or single-block) information model as a graphical sequence logo that reveals conserved sequence patterns and their corresponding gap histogram of spacer lengths. A table is also produced that indicates the individual information contents of each of the binding sites and other important model characteristics.

## Implementation

### Web input

The program is run once all required parameters are specified and sequences are either entered directly or uploaded from a file. Results are either sent via email or are generated on-the-fly. A detailed description, a web snapshot and sample datasets are available on-line [1].

#### Registration

The Bipad server can be accessed in either guest or registration mode. Guests are required to enter a valid email address in order to receive results, since individual information theory is patented. Graphical output (sequence logo and gap histogram) is produced only for registered users. A project title is required, which is also used to label the sequence logo of the binding site alignment. Unless explicitly specified, text output is sent by electronic mail.

#### Search pattern and alignment mode

A search pattern is defined in the most general sense. The search pattern can be either a single-block or bipartite motif which is specified by the lengths of the conserved sequence elements and lengths of the sequences, if any, which separate them. The sequence models may be constrained by specifying that the search identify either one site per input sequence (OOPS) or zero or one sites per sequence (ZOOPS). The respective half-site motifs may be homogeneous (consisting of perfect or imperfect repeats of the same sequence) or heterogeneous (containing different motif widths and/or sequence patterns). The configuration of a bipartite pattern may have four potential half-site orientations: direct repeat (DR), reversed DR (RDR), inverted repeat (IR), and everted repeat (ER).

The Bipad program can search for all possible orientations on either a single strand (DR) or on both strands (DR, RDR, IR, ER). The alignment mode should be specified based on biological evidence or hypothesis, for example, structural or experimental evidence that a protein contacts sequences on a single or both strands. Searches for the best alignments of sequences on both strands are slower than those involving a single strand.

The motif width for a single-block search is specified in the first box of the site width field. It is recommended that a variety of motif widths be explored based either on functional binding site laboratory data or empirical methods (see discussion below;[9]). If the optimal bipartite search pattern is requested, the left- and right-half motifs are specified in the first and second fill-in boxes, respectively. The minimum and maximum gap lengths are also respectively entered in adjacent fill-in boxes.

**Figure 1:**
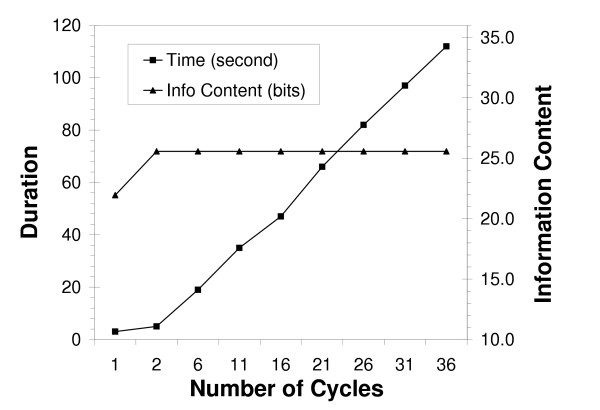
**Performance of Bipad server for alignment of Scaffold/Matrix attachment regions sequences**. Performance of Bipad server for alignment of Scaffold/Matrix attachment region (S-MAR) sites. The graph indicates the linear relationship between cycles and time required for convergence on the optimal model (filled squares), and that relationship between cycles and total information content is asymptotic at two cycles (filled triangles).

Typical searches for bipartite patterns require about twice as long as single-block motifs having the same total width. Note that a single-block is equivalent to zero-gap bipartite pattern, in which two motifs are merged together. Lengthy gaps or broad gap length ranges can increase the time required to determine bipartite motifs, but in most instances, program completes within a minute (Figure 1). The default parameters for half-site and gap length widths have been restricted in order to ensure reasonable multiuser server performance. Half-site widths are permitted to range between 4 to 500 nucleotides in length. Gap lengths between half sites can range from 0 to 200 nucleotides.

#### Number of cycles

In a single Monte Carlo cycle, it is possible for the bipartite local alignment to converge to a local optimum. However, large datasets, searches for subtle motifs characterized by low average information, and bipartite alignment on both strands each require additional cycles to ensure that the preferred alignment will be produced. Increasing the number of cycles increases the confidence that results obtained are the best alignment achievable with this algorithm, at the expense of only a modest drop in server performance (Figure 1). A 500 cycle limit is imposed on model building due to finite server capacity.

The relationships between cycle number and run time, and between cycle number and total information content are illustrated by the bipartite alignment of chromatin scaffold/matrix attachment region binding sites (Figure 1). Increases in cycle number are correlated linearly with the time needed to find the optimal alignment. In this instance, two cycles were sufficient to determine the best bipartite alignment, and further cycling was unnecessary. This default parameter is set to 10 cycles, however it is recommended that a variety of cycling criteria be tested to ensure that a stable solution is obtained.

#### DNA sequences

FASTA-formatted DNA sequences are entered in the sequence field text box or files can be uploaded. Only unambiguous lower or upper case nucleotide symbols are permissible {A, C, G, and T}. Each sequence may be up to 5 kb in length and the number of input sequences is limited to a maximum of 2,000.

#### Refinement

Model refinement is not performed unless this option is specifically requested. The refinement procedure batch executes a series of runs over the specified range of site and gap length widths. The procedure outputs a unit information incremental (UII) plot, which facilitates comparison of information contents gained among a series of potential binding site models. The best model achievable with the Bipad algorithm is the one exhibiting the maximum UII[9].

The specified range of core motif widths should be guided by experimental binding site evidence, if available. Generally speaking, a range of half-site widths averaging 5-mer length half-site would be a reasonable starting point to perform multiple trials of many DNA-protein interactions. Input site lengths shorter than 4 nucleotides are more likely to generate false positive motifs and generally do not represent biologically meaningful binding sites [13]. Very high sequence conservation across a putative binding site warrants further exploration of a wider range of binding site motifs in order to mitigate against the possibility that the model may be truncated at either end. False positive motifs with high information contents may be mitigated by eliminating or pre-filtering, recurrent, low-complexity tandem repeated sequences, if possible, from input sequences [2]. This is particularly important for sets of eukaryotic intergenic non-coding sequences which frequently contain runs of often imperfect homopolymeric sequences that produce minimum entropies that may not be relevant to protein binding.

#### Other options

The lengths of sequences flanking a motif, and the output delivery method may be specified. Flanking nucleotides are displayed in lower case and motifs are shown as upper case. If the specified flanking sequences extend beyond the boundaries of the input sequence, a dash is indicated at those positions. By default, flanking nucleotides are not displayed.

Constraints on the number of aligned DNA sequences, the maximum number of Monte Carlo cycles, maximum half-site widths and gap lengths can be relaxed upon request.

### Web output

Results can be optionally displayed either on-line or sent to the user by electronic mail. If results are output via electronic mail (default), the *bipad-mailer.pl *program sends the bipad output text file to the destination specified in the username field. This file contains: (1) the search parameters and minimum entropy after search; (2) information weight matrix or matrices and a separate frequency matrix formatted for bipartite logo plotter (see below); (3) gap length distribution; (4) a list of nucleotide motifs, their sequence coordinates and information contents for each potential binding site; and (5) parameters used to generate sequence logo with *bipad_logo.pl*. The on-line display dynamically produces a sequence logo (single- or two-block [14], generated with the program *seqlogo.pl *from the WebLogo site; [15]) drawn in PDF, PNG and EPS formats. A bipartite logo is produced by inserting a central gap between two half-site motif logos at the zero coordinate. If the central gap length exceeds maximum permissible gap (currently 10-nucleotides due to limitations on logo image size), a 10-nucleotide gap will be displayed. In addition, we provide an auxiliary sequence logo plotting function to display motif using pre-aligned or user-defined matrices (see below).

Bipartite sequence models [2] dynamically generate a histogram in both graphic and text format that corresponds to the lengths of gaps between half-sites. In addition, a motif table is generated as part of the bipad text output which displays the names of the sequences taken from the FASTA input and the corresponding half site and total individual information contents for each site.

#### Auxiliary function: Bipartite sequence logo plotter

Given either a single frequency matrix or two half site matrices (termed "first" and "second"), the plotter will draw the corresponding sequence logos. The plotter is capable of performing several operations on the original matrices including transforming the "first" matrix by reverse complementation, transforming the "second" matrix through the same operation, transformation of "both" or "none" of the matrices. Only untransformed ("none") and "first" matrix transformation operations can be carried out on single-block matrices. The central gap length of the sequence logo may be specified for bipartite matrices, however the default size is 4 bp. Other options, ie. logo name and size, can also be defined by the user. Detailed input instructions and a working example can be found at [16]. The bipartite output file also includes a separate frequency matrix specifically formatted for use with the bipartite logo plotter.

## Results and discussion

### Case studies

**Figure 2:**
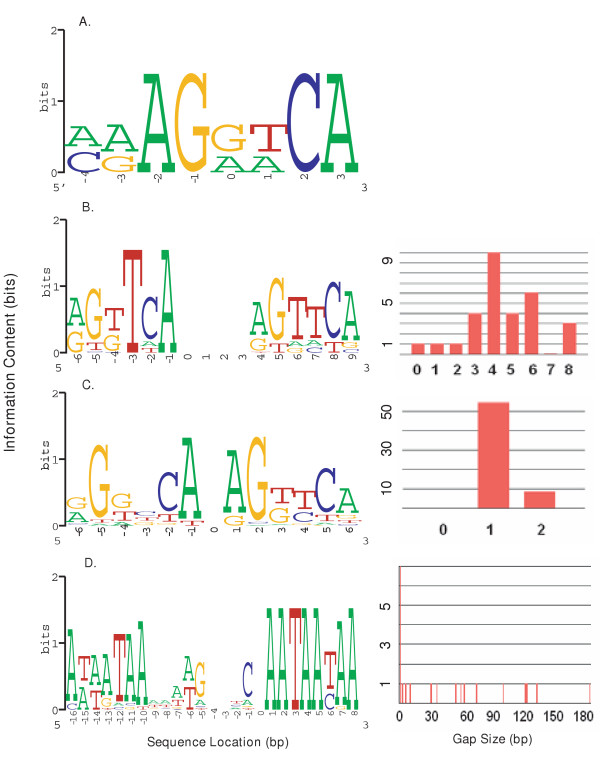
**A gallery of sequence logos**. For bipartite logos, the companion gap histogram is shown on the right. (A) FTZ-F1α monomer binding site; (B) CAR/RXRα PBREM sites; the right-half motif starts at position 4 and positions 0–3 correspond to the central gap between the half-sites; (C) HNF4α homodimer binding sites; the right-half motif starts at position 1, with the variable length gap placed at position 0; (D) MRS bipartite binding sites; the second half-site motif begins at position 1; the variable length gap denoted by the distribution corresponds to position 0 of the logo. Corresponding Bipad text file output for these models can be viewed at [1].

To illustrate results produced by the server, we analyzed single-block and bipartite sequences recognized by several DNA-binding proteins (Figure 2). Hormone responsive elements (HRE's) may be recognized by nuclear hormone receptors which bind as monomers (FTZ-F1α), homodimers (HNF4α) and heterodimers (CAR/RXRα). The server was also used to compute models of chromatin matrix attachment regions (S/MAR) which are composed of heterogeneous bipartite binding elements. Bipartite models for the same datasets generated with either the OOPS or the ZOOPS parameter produced identical alignments. The unaligned sequences used in the preparation of these models are available on the Bipad website.

FTZ-F1α (Figure 2A) is an orphan nuclear receptor known to bind as a monomer to HREs containing the consensus sequence, TCAAGGTCA. Expression of FTZ-F1α occurs in precursors of adrenal steroidogenic tissue and gonadal steroid-producing cells [17]. Experimentally verified monomeric binding sequences [18] were aligned with Bipad. The motif width was set as 8 bps on forward strand and one cycle was run to find the motif, as this is a small dataset in which motif length is equal to the sequence length. The average information content is 8.78 bits per site. The single-block sequence logo is indicated in Figure 2A.

Figure 2B indicates a bipartite model based on recognition sites bound by the nuclear receptor constitutive androstane receptor (CAR), which forms a heterodimeric complex with the retinoid X receptor (RXRα) that binds to phenobarbital-responsive elements (PBREM) of target genes to regulate their expression [19]. CAR/RXRα recognizes a degenerate PBREM sequence consisting of a bipartite pattern of two half-sites with separated by flexible nucleotide spacer. Our bipartite algorithm is well suited for modeling CAR/RXRα sites, as the heterodimer has been shown to recognize DR, RDR, IR and ER patterns [19]. The sequences of 30 human CAR/RXRα binding sites were extracted from the *CYP3A4, CYP3A7, CYP2C9, CYP2C19, CYP2B6, UGT1A1, MRP2 *and *iNOS *genes and aligned. Alignment of half sites on both strands was permitted, consistent with published binding studies indicating that all possible orientations should be considered. The half-site and gap range lengths were set to 6<[0, 8]>6 (see below for a refinement procedure). A single cycle was needed to find the best alignment. The model has an average information content per bipartite site is 13.87 bits and the degenerate patterns discovered are consistent with the experimentally verified sites (RKKTCA<0–8>RKKTCA) [19]. Analysis of the same set of binding sites with BioProspector [8] produced a similar alignment; however the logo contained only 12.9 bits because conserved half sites present on both strands were not detected.

HNF4α (Figure 2C) binds as a homodimer to DR HREs separated by one or two nucleotides (DR1, DR2). HNF4α was initially identified as a transcription factor required for liver-specific gene expression, and later was shown to be expressed high level in liver, kidney, intestine, and pancreas and at low levels in the testis [17]. The average information model is based on 63 validated binding sequences and flanking sequences that have been collated from multiple genes and species [20]; (F. Sladek, personal communication). Due to limited available flanking sequence and experimental observation, the bipartite search pattern was constrained to 6<[1, 2]>6 and the optimal bipartite alignment was found in a single Monte Carlo cycle. Assuming that all orientations could be bound, nearly all of the sites identified by Bipad were DR. The average information content per bipartite site is 11.23 bits. The discovered patterns are consistent with the experimentally verified sites [20] and those produced by BioProspector.

In Figure 2D, genomic elements of Scaffold/Matrix attachment regions (S/MAR), which delineate structural and functional organization in eukaryotic genomes[21,22], are modelled. A bipartite sequence element associated with S/MARs has been reported based on sequences of 23 bipartite elements from different species (chicken, Chinese hamster, *Drosophila*, rabbit, yeast, SV40, human and mouse). The resultant model is expected to detect a highly conserved subset of potential S/MAR elements that are common to these and related species. The sequences containing potential sites were embedded on the same strand in an average human genomic background (G and C content are 21%, and A and T content are 29%, respectively) to form sequences 250 nucleotides in length, allowing for a large range of potential inter-half site distances. Five overlapping sites separated by 1-bp gaps were embedded, since Bipad is not configured to handle overlapping binding sites. The proposed matrix attachment region recognition signature (MRS) is represented by a pair of degenerate asymmetric half-sites (often containing the sequences: AATAAYAA and AWWRTAANNWWGNNNC), separated by a nucleotide spacer of up to 200 bp in length. The bipartite search pattern was set to 16<[0, 200]>8 on the forward strand and 2 cycles was sufficient to locate all the embedded MRS sites, except for two left half-sites. In the first case, the half-site is shifted 6 nucleotides away from its original location and has higher information content (13.615 bits) than the embedded sequence (9.819 bits), whereas the second left half-site is 81 bps downstream of the implanted motif and its information content is very similar to that of the original site (11.5 bits). For this reason, the aligned sequence motif has, on average, slightly more information (12.2 bits) than the experimentally determined sequence (12.0 bits) for left-half site. The total average information content per MRS site is 25.8 bits, with the right-half site being more conserved (13.6 bits) than the left-half (12.2 bits). Thus, the right-half is somewhat more highly conserved than the left-half site (Figure 2D). The sequence logo reveals that the model to be more heterogeneous than the published MRS consensus sequence and the half site sequence patterns are somewhat different.

#### Refinement

**Figure 3:**
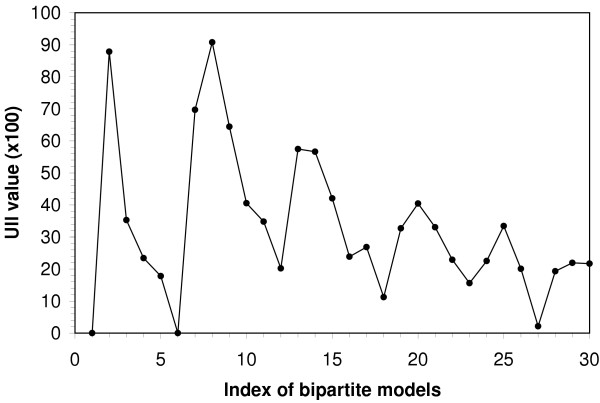
**Refinement of CAR/RXR bipartite binding motif models**. The x-axis represents an index of binding sites models of increasing site widths beginning with the initial input parameters defining site width and gap range were 5<[0,8]>5. For example, Model 1 corresponds to the motif pattern: 5<[0,8]>5, Model 2 is 5<[0,8]>6; where the final model, number 30, corresponds to the pattern 10<[0,8]>9. The unit incremental information (UII) value is computed for each motif and displayed on the Y-axis. The maximum UII usually has the highest information density and is indicative of the optimal model.

Figure 3 shows the progressive refinement of the bipartite CAR/RXRα binding motif models. To enable this program function, the 'refine' option is selected and the initial or basis search pattern is defined. The program generates and evaluated models with left and right motifs of increasing lengths. Treating 5<[0,8]>5 as the basis motif, we calculate the UII value for each motif. The UII plot orders these models along the X-axis, with model 1 corresponding to the motif pattern: 5<[0,8]>5, Model 2 to 5<[0,8]>6, through Model 30, which has the pattern, 10<[0,8]>9. The 6<[0,8]>6 motif (Figure 3) displays the highest information increment (see bipartite logo in Figure 2B), ie. the highest level of information density (bits per unit length) over all of the motifs analyzed, making it arguably the optimal binding site model.

#### Performance vs. sequence length

**Figure 4:**
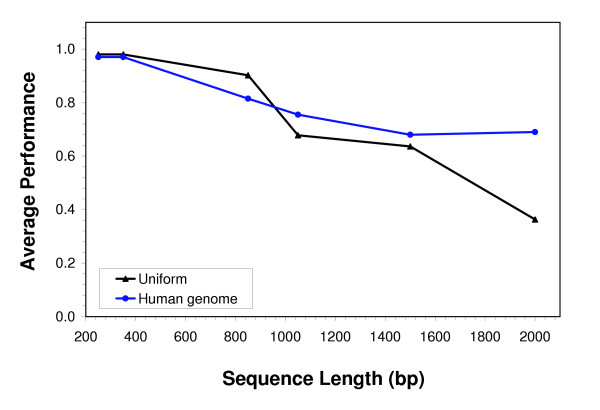
**Bipad performance for various input sequence lengths**. The graph shows the performance of Bipad (Y-axis) for recognition of S-MAR binding sites embedded in background sequences of varying lengths. Each S-MAR site was embedded in a background either with a uniform composition [black line], or having the average human genomic composition [blue line]. The background sequence was varied from 250 to 2000 bp in length (X-axis). The performance calculation is given in Reference [2]; each data point has been averaged over three replicates.

To examine the performance of Bipad for detection of true binding sites in sequences of varying lengths, we embedded the MRS binding sites in background sequences having either a uniform equiprobable nucleotide distribution or an average human genomic composition (described above). We embedded exactly one MRS site in each background sequence (23 sequences in total) and varied the lengths of each of the background sequences from 250 bp to 2000 bp (repeated three times for each such simulation). The average performance (based on detection of the embedded sequence) in each group is shown in Figure 4. For sequences less than 1 kb in length, binding sites were detected with accuracy of over 80% regardless of background composition; however, as the sequence length increases, the performance decreases monotonically. It is interesting to note, however, that MRS sites embedded in a background having a composition similar to that of the human genome were more easily detected in longer sequences (e.g. 2 kb) compared to sites embedded in uniformly-distributed background.

## Conclusion

The Web service presented here can be used for either *a priori *detection or *ab initio *discovery of single-block or bipartite binding sites. The examples provided demonstrate that Bipad can be broadly applied to many different types of motifs, regardless of their level of sequence conservation [2]. We evaluated server performance by constructing models of published binding sites for several transcription factors and chromatin binding proteins. The motif sites found by the Bipad server are consistent with sequences that have been experimentally identified as binding sites. However, a domain-specific understanding of the protein-nucleic acid interactions for particular protein is essential in selecting realistic parameters (site lengths and orientations) that take advantage of Bipad's capabilities. Site information contents predicted by Bipad are related to their corresponding binding affinity and can be experimentally validated. By interactively exploring various pattern lengths and orientations, the web server efficiently provides reasonable computational models for experimentally-validated binding site data.

The Bipad algorithm assumes zero or one bipartite site to be present in each training sequence. Bipad does not utilize multiple degenerate TFBS recognized by the same factor in a single sequence; to include all experimentally validated sites in the same promoter in a bipartite model, intervals containing individual TFBS should be separated into different input sequences.

The software was originally designed and implemented for localizing nuclear receptor binding sites that are often bipartite patterns, some containing half-sites in all possible orientations. However, the program can be used to efficiently identify single-block motifs as well [2], as this is a special case of bipartite motif definition.

We plan to extend Bipad for large-scale genomic sequence analysis, however this task will be challenging. Although many tools for discovery of TFBS elements have been developed, a comprehensive solution that accurately defines binding sites in genomic sequences has been elusive for a variety of reasons [23]. The known limitations in computational methods cannot be overcome until several significant laboratory-derived problems are addressed. Collections of binding sites recognized by the same protein are known to exhibit pervasive systematic bias [12]. As we have shown, inadequate localization of binding sites in sequence data from chromatin immunoprecipitation assays can compromise accurate detection of subtle binding site signals. Finally, false positive binding sites can be introduced through microarray- derived artifacts in ChIP-chip hybridizations[24]. A complete and accurate biological understanding of DNA-protein interactions is a prerequisite to the accurate identification of binding sites in long genomic sequences.

## List of abbreviations

Bipad – Bipartite pattern discovery

## Availability and requirements

• **Project name: **Modeling Bipartite cis-elements, Bipad

• **Project home page: **

• **Operating system(s): **Platform independent

• **Programming language: **C++ and Perl

• **Other requirements: **None

• **License: **see:

• **Use restrictions for non-academics: **see 

## Authors' contributions

CB and PKR conceived of the project, and CB designed and implemented the algorithms. Both authors wrote the manuscript.
